# Pregnancy-Induced Atypical Hemolytic Uremic Syndrome

**DOI:** 10.7759/cureus.90111

**Published:** 2025-08-14

**Authors:** Alexander Den Boef, Sneh Parekh, Melville C O'Brien, Shiguang Liu, Rafik Jacob

**Affiliations:** 1 Department of Internal Medicine, University of Florida College of Medicine – Jacksonville, Jacksonville, USA; 2 Department of Pathology and Laboratory Medicine, University of Florida College of Medicine – Jacksonville, Jacksonville, USA

**Keywords:** anemia, atypical hemolytic-uremic syndrome (ahus), eculizumab, hemolytic uremic syndrome, pregnancy, pregnancy-associated atypical hemolytic uremic syndrome, renal dysfunction, thrombocytopenia

## Abstract

Atypical hemolytic uremic syndrome (aHUS) is a rare form of thrombotic microangiopathy that causes anemia, thrombocytopenia, and acute renal failure. In aHUS, a triggering event can lead to dysregulation of the complement immune system, leading to a cascade of events resulting in clot formation, capillary thrombosis, and renal dysfunction. Numerous triggers for aHUS exist, and pregnancy-associated aHUS (P-aHUS), in particular, is associated with poor maternal outcomes, with high risk for adverse events due to the inherent difficulties in establishing the diagnosis and treatment, along with the sequelae associated with the disease itself. We present a case of P-aHUS here, highlighting the diagnostic challenges, clinical management, and the importance of timely intervention, while also emphasizing the role of the monoclonal antibody drug eculizumab in its treatment.

## Introduction

Atypical hemolytic uremic syndrome (aHUS) is a relatively rare form of thrombotic microangiopathy (TMA) resulting in the destruction of red blood cells, thrombocytopenia, and acute renal failure. The annual incidence of aHUS is estimated to be between 0.23 and 0.42 cases per million population [1]. While research and literature exist regarding aHUS, its unique pathophysiology and variations in gene and complement pathways are not yet fully understood and are still being researched, with new mechanisms of pathogenesis still being identified [1]. In aHUS, it has been proposed that a triggering event causes activation of a genetic mutation or autoantibody, which causes dysregulation of the complement immune system, leading to clot formation, capillary thrombosis, and glomerular wall thickening. Numerous triggers for aHUS exist, including cancer, infection, certain medications, and pregnancy. Pregnancy-associated aHUS (P-aHUS) in particular is associated with poor maternal outcomes, with a high risk for adverse events.

In pregnancy, the postpartum period is recognized as a potential trigger for the onset of aHUS. Although rare, P-aHUS accounts for approximately 7-20% of all aHUS cases and typically presents in the third trimester or within days to weeks after delivery [1]. Differentiating P-aHUS from other pregnancy-related TMAs, such as preeclampsia, hemolysis, elevated liver enzymes, low platelet count (HELLP) syndrome, and thrombotic thrombocytopenic purpura (TTP), poses a diagnostic challenge due to overlapping clinical features [2,3]. Additionally, no direct diagnostic test for identification of aHUS exists [1].

Early recognition of P-aHUS is crucial, as delayed diagnosis and treatment are associated with poor renal outcomes and a lifelong risk of multiorgan complications of TMA [4]. The introduction of monoclonal antibodies such as eculizumab, acting as a complement inhibitor, has significantly improved outcomes by targeting underlying complement dysregulation [4]. Here, we present a case of P-aHUS, highlighting the diagnostic challenges, clinical management, and the importance of timely intervention.

This article was previously presented as a meeting abstract and poster at the 2025 Southern Regional Meeting on February 13, 2025.

## Case presentation

We present the case of a 33-year-old female patient, gravida 2, para 2, with a past medical history of gestational hypertension, alcohol and tobacco use, and recent cesarean delivery, who initially presented for right-sided abdominal and back pain. Since her cesarean delivery 12 days before, she reported worsening lower extremity swelling and increased fatigue, with progressively decreasing urine output. On physical examination, the patient had sharp, right-sided costovertebral angle tenderness that radiated to her groin and 1+ pitting edema of her right lower extremity. Initial vital signs were significant for a respiratory rate of 26 breaths/minute and a blood pressure of 141/85 mmHg. Following admission, her kidney function continued to worsen with an associated thrombocytopenia and anemia for which nephrology and hematology oncology were also consulted.

Laboratory testing was remarkable for a stage 3 acute kidney injury, elevated N-terminal prohormone of brain natriuretic peptide, normocytic anemia, and thrombocytopenia (Table 1). Further workup in the form of hemolysis testing (lactate dehydrogenase, LDH, and haptoglobin) returned positive, a urinalysis returned positive for blood, and autoimmune and infectious testing returned negative.

**Table 1:** Relevant laboratory testing obtained IU: international units; HPF: high-power field

Relevant laboratory testing	Value	Reference ranges
Complete metabolic panel		
Sodium	136 mmol/L	135-145 mmol/L
Potassium	3.6 mmol/L	3.3-4.6 mmol/L
Chloride	103 mmol/L	101-110 mmol/L
Carbon dioxide	18 mmol/L	21-29 mmol/L
Urea nitrogen	35 mg/dL	6-22 mg/dL
Creatinine	3.31 mg/dL	0.51-0.96 mg/dL
Glucose	91 mg/dL	71-99 mg/dL
Calcium	8.2 mg/dL	8.6-10 mg/dL
Total protein	5.7 g/dL	6.5-8.3 g/dL
Anion gap	15 mmol/L	4-16 mmol/L
Estimated glomerular filtration rate	18 mL/minute/1.73 m^2^	≥60 mL/minute/1.73 m^2^
Complete blood count		
White blood cells	9.49 × 10^3^/µL	4-10 × 10^3^/µL
Red blood cells	2.68 × 10^6^/µL	4-5/2 × 10^6^/µL
Hemoglobin	7.8 g/dL	12-16 g/dL
Hematocrit	24%	35-45%
Mean corpuscular volume	89.6 fL	78-100 fL
Platelet count	128 × 10^3^/µL	150-450 × 10^3^/µL
Lactate dehydrogenase	1,766 IU/L	135-214 IU/L
Haptoglobin	<10 mg/dL	40-200 mg/dL
Autoimmune labs		
Antinuclear antibody	Negative	Negative
Cytoplasmic antineutrophil cytoplasmic antibody	<1:20	<1:20
Perinuclear antineutrophil cytoplasmic antibody	<1:20	<1:20
Atypical perinuclear antineutrophil cytoplasmic antibody	<1:20	<1:20
Coagulation		
Anticardiolipin IgA, IgG, and IgM	Negative	Negative
Beta-2 glycoprotein 1 IgA, IgG, and IgM	Negative	Negative
Lupus anticoagulant	Negative	Negative
Fibrinogen	742 mg/dL	146-461 mg/dL
International normalized ratio	1	0.8-1.1
Urinalysis		
Color	Red	Amber
Blood	Large	Negative
Glucose	Negative	Negative
Nitrite	Negative	Negative
Leukocyte esterase	Negative	Negative
Protein	≥1,000 mg/dL	0 mg/dL
White blood cells	7 HPF	0-5 HPF
Red blood cells	6 HPF	0-5 HPF
Bacteria	Rare	None seen
Hyaline casts	Present	None seen
Miscellaneous testing		
Hepatitis B surface antigen	Negative	Negative
Hepatitis C antibody	Negative	Negative
HIV Ag/Ab, fourth generation	Nonreactive	Nonreactive
C3 complement	207 mg/dL	90-180 mg/dL
C4 complement	51 mg/dL	10-40 mg/dL
ADAMTS13	85.4%	>60%
Shiga toxin assay	Negative	Negative

**Figure 1:**
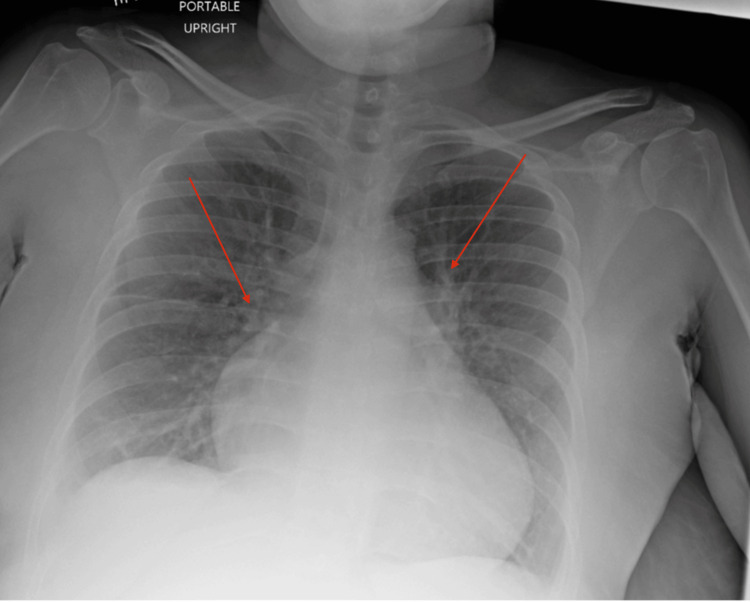
Chest X-ray demonstrating cephalization of the pulmonary vasculature with vascular engorgement and mild interstitial pulmonary edema (red arrows)

On imaging, a chest X-ray showed mild interstitial pulmonary edema with vascular engorgement (Figure 1).

**Figure 2:**
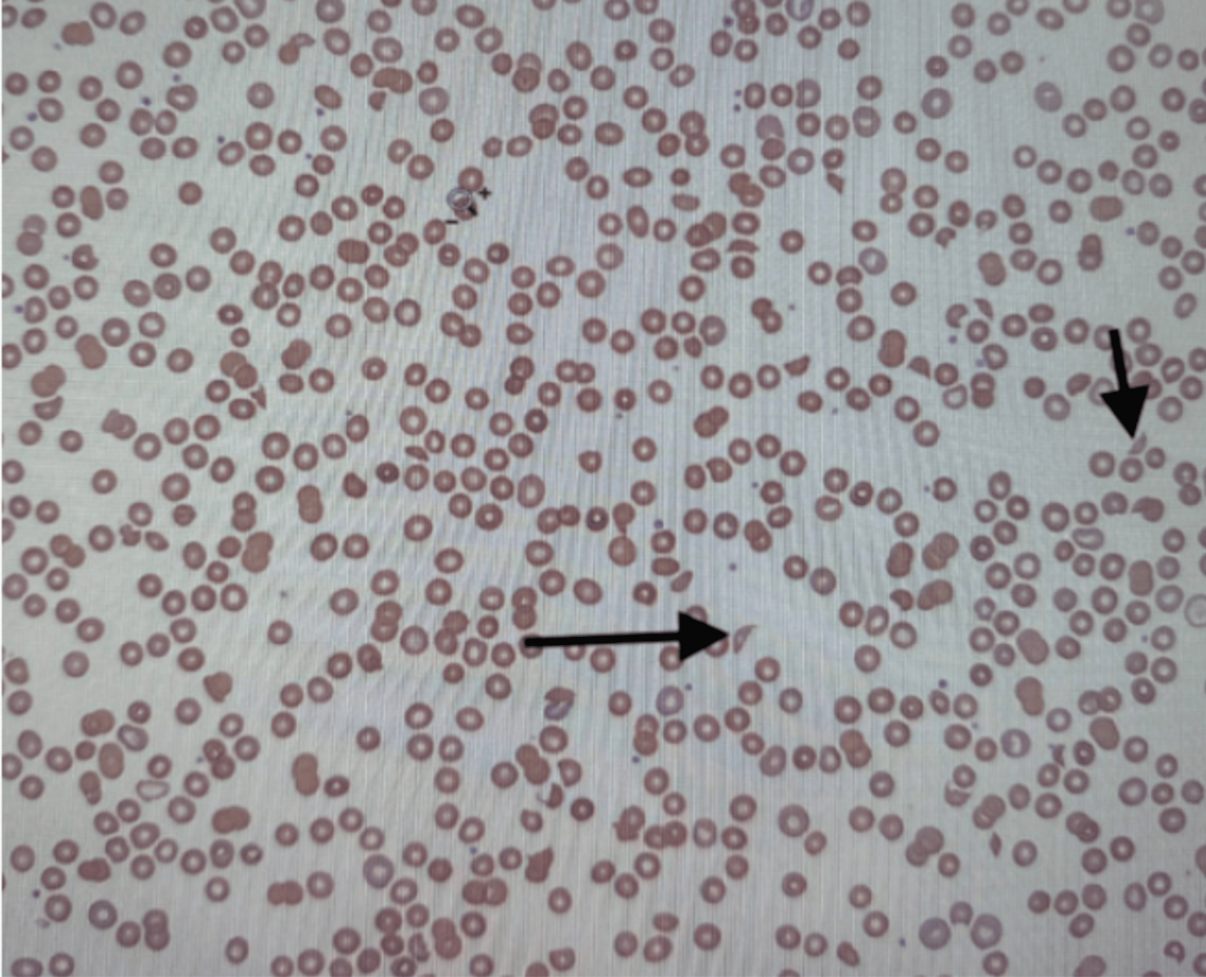
Peripheral blood smear showing schistocytes (arrows)

Following initial laboratory workup indicative of hemolysis, with elevated LDH, decreased haptoglobin, and persistent drops in her hemoglobin, requiring multiple blood transfusions, she also began to have rapidly deteriorating renal function, eventually requiring hemodialysis. As such, the peripheral blood smear was examined, where schistocytes were seen, consistent with hemolytic anemia (Figure 2).

**Figure 3:**
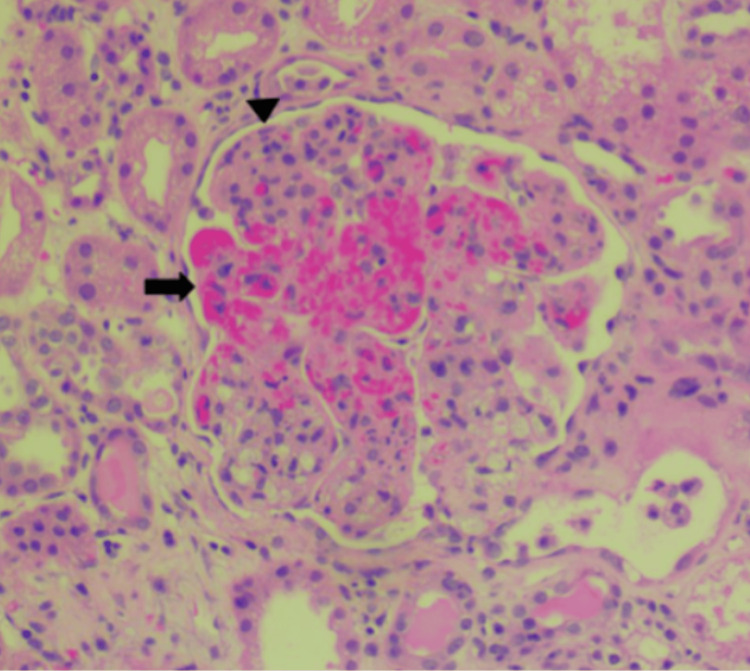
Kidney biopsy glomerulus with fibrin thrombi (seen by the arrow) and endothelial swelling (seen by the arrow head) Performed via H&E stain 100× H&E: hematoxylin and eosin

A kidney biopsy was then performed, showing acute tubular injury with glomerular and arteriolar fibrin thrombi, consistent with TMA (Figure 3).

In summary, initially, after the surgery, the patient began to have a progressive decline in her urine output. She was postoperative day 11 following her cesarean section when she began to have the symptomatic onset of right-sided abdominal and back pain. Laboratory values that day were indicative of an acute kidney injury with an elevated creatinine, increased from her noted baseline of 0.58 mg/dL, and a downtrend in her hemoglobin from 8.6 g/dL, both previously recorded on postoperative day 2. On postoperative day 12, the patient also started to have associated right-sided flank pain, after which she was transferred to our internal medicine service. By postoperative day 14, given her initial laboratory workup, as seen above in Table 1, consistent with hemolysis, thrombocytopenia, and a progressively worsening acute kidney injury, her hemoglobin continued to drop requiring intermittent red blood cell transfusions, with a worsening thrombocytopenia, and an increasing creatinine, respectively, after which nephrology and hematology oncology were consulted. On postoperative day 15, the patient underwent a renal biopsy, with pathology indicative of microvascular thrombi. In conjunction with the normal ADAMTS13 activity, the diagnosis of complement-mediated TMA and aHUS was made. On postoperative day 16, the patient was initiated on hemodialysis.

Numerous differential diagnoses were considered in the evaluation of this patient. Given her pregnancy, with associated sudden-onset and progressively worsening hemolytic anemia, the differential diagnosis at this time included pathologies such as HELLP syndrome, TTP, and Shiga-toxin-associated hemolytic uremic syndrome (STEC-HUS). After performing the laboratory workup and obtaining the full results of testing, a normal ADAMTS13 activity and negative Shiga toxin ruled out TTP and STEC-HUS, respectively. Given the clinical picture and exclusion of alternative diagnoses, complement-mediated aHUS was strongly suspected. Complement gene testing was sent; however, it returned negative.

While her complement testing was unrevealing, the clinical picture and renal biopsy findings, showing evidence of TMA, all strongly supported a diagnosis of aHUS. Due to high suspicion for aHUS, per management and treatment guidelines, the patient received prophylactic meningococcal vaccination and empiric ceftriaxone, in preparation for treatment with a monoclonal complement inhibitor. Eculizumab, a monoclonal antibody targeted against complement C5, was initiated; however, administration was initially delayed due to the expense of the drug and the patient’s associated insurance authorization issues. Upon initiation, the patient received her first two weeks of eculizumab treatment on postoperative days 18 and 25, one week apart, during which the patient experienced gradual improvement with noted stabilization in laboratory values upon discharge. Given her symptomatic improvement, the patient was then discharged with planned outpatient follow-up during which she was instructed to complete her eculizumab treatment, and has since noted improvement of kidney function and overall clinical status.

## Discussion

aHUS is a rare complement-mediated TMA that often presents with microangiopathic hemolytic anemia, thrombocytopenia, and acute kidney injury. In this case, the patient developed classic features of aHUS in the postpartum period, including schistocytosis, elevated LDH, low haptoglobin, anemia, thrombocytopenia, and worsening renal function requiring dialysis. The presence of fibrin thrombi in the renal biopsy confirmed a diagnosis of TMA. Through laboratory testing, other etiologies such as TTP, Shiga-toxin-producing *Escherichia coli* HUS (STEC-HUS), and HELLP syndrome were ruled out based on negative ADAMTS13 activity, Shiga toxin testing, and the timing of symptom onset with the associated clinical presentation, respectively.

Pregnancy and particularly the postpartum period are recognized triggers for aHUS due to the physiological activation of the complement system during pregnancy and delivery. Approximately 79% of P-aHUS cases occur after delivery [1]. Our patient’s disease onset shortly after cesarean section is consistent with this pattern. Her prior history of gestational hypertension is likely to have predisposed her to further postpartum complications and may have further primed the endothelium for complement-mediated injury.

The absence of mutations in complement-regulatory genes does not exclude a diagnosis of aHUS, as up to 40-50% of cases may lack identifiable mutations [5,6]. In this case, complement testing was unrevealing, yet the clinical picture and biopsy findings all strongly supported aHUS. Prompt recognition and treatment are crucial, as delayed therapy is associated with poor renal outcomes. Eculizumab, a monoclonal antibody that inhibits terminal complement component C5, has dramatically changed the prognosis of aHUS, allowing for hematologic remission and recovery of renal function in many cases [4]. By inhibiting terminal complement activation, eculizumab halts the progression of endothelial injury and microvascular thrombosis [5]. Clinical trials and real-world studies have demonstrated that early initiation of eculizumab in aHUS leads to rapid normalization in hematologic values, improvement in renal function, and decreased rates of dependence on dialysis [7-9]. According to a retrospective study by Bruel et al., 56% of patients with P-aHUS achieved independence from dialysis, with better noted outcomes among those who received eculizumab sooner [3]. In our patient, the initiation of eculizumab therapy led to the resolution of hemolysis, improvement in platelet counts, and gradual recovery of kidney function, emphasizing its role as a first-line therapy in suspected aHUS.

Although the optimal duration of eculizumab therapy in aHUS remains under debate, current expert guidelines recommend at least 6-12 months of treatment, with ongoing monitoring for relapse [6]. Additionally, it is also important to note that since eculizumab increases susceptibility to meningococcal infections, appropriate vaccination and prophylactic antibiotics are necessary before or during therapy initiation, as was the case in our patient. Finally, as eculizumab is a monoclonal antibody, the medication is associated with high costs and can serve as a financial barrier to early initiation. A single vial of the medication can exceed 6,000 USD, with yearly maintenance costs exceeding hundreds of thousands of dollars, and as such, biosimilars to eculizumab are also now being studied for their potential benefits and positive outcomes in the treatment of aHUS [10].

## Conclusions

P-aHUS is a rare but serious complication that requires high clinical suspicion, particularly in postpartum patients presenting with anemia, thrombocytopenia, and renal dysfunction. This case highlights the importance of distinguishing aHUS from other TMAs and pregnancy-related conditions such as HELLP and preeclampsia. Kidney biopsies and the exclusion of TTP and STEC-HUS are vital for a definitive diagnosis. Early treatment with eculizumab can lead to significant recovery and improved outcomes. Clinicians should remain vigilant for this life-threatening condition in postpartum patients with sudden-onset hemolysis and multisystem involvement.
